# Spittlebug damage on tropical grass and its impact in pasture-based beef production systems

**DOI:** 10.1038/s41598-020-67490-9

**Published:** 2020-07-01

**Authors:** Guilhermo Francklin Souza Congio, Pedro Castro de Almeida, Tadeu Ruzza Barreto, Vitor Afonso Tinazo, Tiago Alves Corrêa Carvalho da Silva, Diogo Fleury Azevedo Costa, Moacyr Corsi

**Affiliations:** 1 Colombian Corporation for Agricultural Research, Agrosavia, Km 14 via Mosquera, Bogota, Cundinamarca 250047 Colombia; 2 Animal Science Department, “Luiz de Queiroz” College of Agriculture (USP/ESALQ), University of Sao Paulo, Av. Padua Dias, 11, Piracicaba, Sao Paulo 13418-900 Brazil; 3 School of Environmental and Rural Science, University of New England, Armidale, NSW 2351 Australia; 4 Centre for Animal Science, Queensland Alliance for Agriculture and Food Innovation, The University of Queensland, Gatton, QLD 4343 Australia

**Keywords:** Plant physiology, Plant stress responses, Biotic, Sustainability, Entomology

## Abstract

Spittlebugs are the main pest of tropical pastures and Marandu palisade grass (*Urochloa brizantha* cv. Marandu) is the most representative cultivated pasture in the tropics. Our objective was to characterize Marandu palisade grass responses subjected to *Mahanarva* (Hemiptera: Cercopidae) attack and to estimate the losses in terms of beef production from pasture-based systems. A set of five experiments were carried out. Three consecutive years of monitoring showed that *Mahanarva* spittlebugs increased their abundance after first rains with three to four peaks throughout the wet season. A decrease of 66% on herbage yield was observed in the greenhouse trial, with an average decrease of 61% on pools of calcium, magnesium, phosphorus, sulfur, potassium, crude protein, neutral-detergent fiber and in vitro digestible dry matter of Marandu palisade grass. Results from field experiments corroborated with greenhouse trial showing decreases on herbage yield varying from 31 to 43% depending on level of fertilization and grazing severity of Marandu palisade grass. Finally, an unprecedented 154-ha field experiment indicated that *Mahanarva* decreases 74% the beef productivity (i.e. kg body weight ha^−1^) of Nellore heifers grazing Marandu palisade grass.

## Introduction

Brazil has the largest commercial beef cattle herd and is one of the top beef exporters in the world^1^. Brazilian beef represents 20% of all global beef trading and, together with other agricultural commodities, it sums to about 21% of gross domestic product in Brazil^2^. Beef cattle production in Brazil is characterized by pasture-based systems of which almost 90% of the cattle slaughtered comes from^3^. However, despite highly representative, pasture-based livestock systems are extensive and conducted with low technology inputs resulting in low stocking rates (SR) and animal performance (i.e. average daily gain, ADG). Studies have reported beef production greater than 1,500 kg of body weight (BW) ha year^−1^ whilst the Brazilian average is much lower at about 150 kg BW ha year^−1^^4^. Many factors stand out as key drivers for this poor productivity, among them: inadequate grazing management strategies, low soil fertility, and pest damage^5^.

The 215 million head of cattle in Brazil occupy approximately 160 million hectares composed by 70% of cultivated pasture species and 30% of native grasslands^6,7^. Marandu palisade grass {*Brachiaria brizantha* (Hochst. ex A. Rich.) R. D. Webster [syn. *Urochloa brizantha* (A. Rich.) Stapf]} is the most representative of the cultivated pasture species, grown in about 50 million hectares in the country^8^. It was launched by Embrapa in 1984 as an alternative to signal grass (*Brachiaria decumbens* cv. Basilisk, syn. *Urochloa decumbens* Stapf R.D. Webster) owing to its higher herbage yield and, mainly because of its resistance to “pasture spittlebugs” such as *Notozulia entreriana* (Berg, 1879), *Deois flavopicta* (Stål, 1854) and *Deois schach* (Fabricius, 1787) (Hemiptera: Cercopidae)^9^. However, from the mid-1990s, spittlebugs from the genus *Mahanarva* have been reported infesting several species of Brazilian tropical pastures, including Marandu palisade grass^10,11^. According to Valério^12^, this spittlebug genus had previously been reported damaging sugarcane (*Saccharum officinarum*). Thenceforth, there had been species such as *M. spectabilis* (Distant, 1909), *M. posticata* (Stål, 1855), *M. liturata* (Le Peletier & Serville, 1825), and *M. fimbriolata* (Stål, 1854) reported damaging pastures in Brazil^13–15^. Despite the recently increase of research on *Mahanarva* in tropical pastures, no study has quantified the damage caused by this genus.

Spittlebugs are considered the main pest of tropical pastures due to their wide distribution and damage capacity^12^. In Brazilian wet-warm tropical condition, the spittlebug occurrence coincides with the wet season occurring usually from September to March (≈ 180 days) and it is affected by the precipitation regime of each region^12,16^. Overall, after the first rains, nymphs eclode from quiescent eggs oviposited in the last wet season^12,17^, then they fix at different host species and constantly suck the sap of roots xylem, meanwhile the adults suck the sap from the leaf xylem and inject a salivary excretion containing toxic enzymes^18,19^. The phytotoxemia is initially characterized by a longitudinal chlorosis along the leaf that evolves to necrosis promoting the burnt aspect and may even cause the death of tillers depending on spittlebug population density^20,21^. The number of generations may vary from 3 to 4 depending on the wet season duration^10,22^.

Damage caused by spittlebugs affects shoot and root development^23,24^. Studies with other sucking pests reported changes on photosynthates allocation pattern 24 h after they start feeding and decrease organic reserve pools^25,26^. Spittlebug damage on forage species includes decreased reserves and root growth^27,28^, reduced shoot regrowth and herbage yield^29–33^, and poor nutritive value of herbage^31,33,34^. However, the magnitude of these damages may be affected of the availability of growth factors^32^. These studies suggest a possible decrease in beef production owing to both decreased SR and ADG caused by lower herbage yield and nutritive value, respectively^5^. In fact, there is only one modeling study that estimated losses widely varying from 5 to 54% depending on infestation level^35^. However, the damage in beef productivity has not yet been measure because there is not a direct effect on animal product (i.e. meat or milk), but on pasture^36^. Therefore, there is knowledge gap regarding the impact of spittlebug damage on beef production from tropical pasture-based systems. The central hypothesis of this study was that spittlebugs from the genus *Mahanarva* can strongly affect Marandu palisade grass physiology depending of soil fertility level and decrease beef productivity from typical pasture-based systems in the Brazilian tropic. Our objective was to characterize and to quantify the responses of Marandu palisade grass subjected to *Mahanarva* attack in contrasting soil fertility levels and to estimate the losses in terms of beef production from pasture-based systems.

## Results

### Greenhouse trial

**Figure 1:**
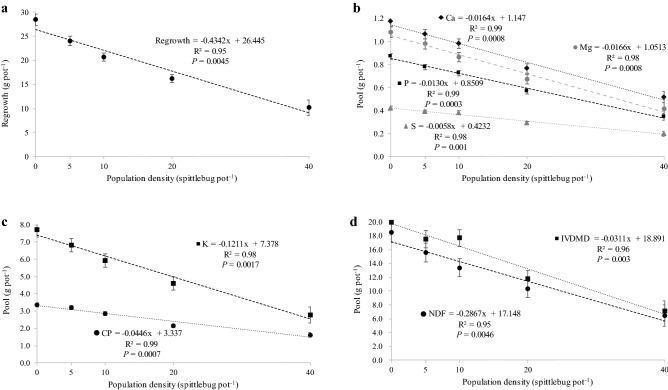
Regrowth mass (**a**) and pools of calcium, magnesium phosphurus and sulfur (**b**), potassium and crude protein (**c**), and in vitro dry matter digestibility and neutral detergent fiber (**d**) of Marandu palisade grass growing in greenhouse and infested by adults of *Mahanarva* spittlebugs at Piracicaba, SP, Brazil. Error bars denote the standard error of the mean.

There was no effect of spittlebug population density on both stubble (*P* = 0.1312) and root mass (*P* = 0.9547). Stubble and root mass averaged 27.7 and 73.6 g pot^−1^, respectively. In addition, the organic reserves from stubble and root were not affected by spittlebug. Pools of non-fibrous carbohydrate (NFC) and nitrogen (N) from stubble averaged 8.5 and 0.27 g pot^−1^ (*P* = 0.0884 and *P* = 0.3183), respectively. On the other hand, pools o NFC and N from roots averaged 25.2 and 0.37 g pot^−1^ (*P* = 0.9416 and *P* = 0.4134), respectively. However, spittlebug population density affected regrowth mass (*P* = 0.0045) with results adjusting a negative linear regression model (Fig. 1a). Forty spittlebug pot^−1^ decreased 66% the regrowth mass compared to check (no spittlebug). Pools of calcium (Ca) (*P* = 0.0008), magnesium (Mg) (*P* = 0.0008), phosphorus (P) (*P* = 0.0003), sulfur (S) (*P* = 0.001), potassium (K) (*P* = 0.0017), crude protein (CP) (*P* = 0.0007), in vitro digestible dry matter (IVDDM) (*P* = 0.003), and neutral detergent fiber (NDF) (*P* = 0.0046) were influenced by *Mahanarva* attack with results adjusting a negative linear regression model (Fig. 1b–d).

### Field experiments

#### Pattern of spittlebug distribution and abundance

**Figure 2:**
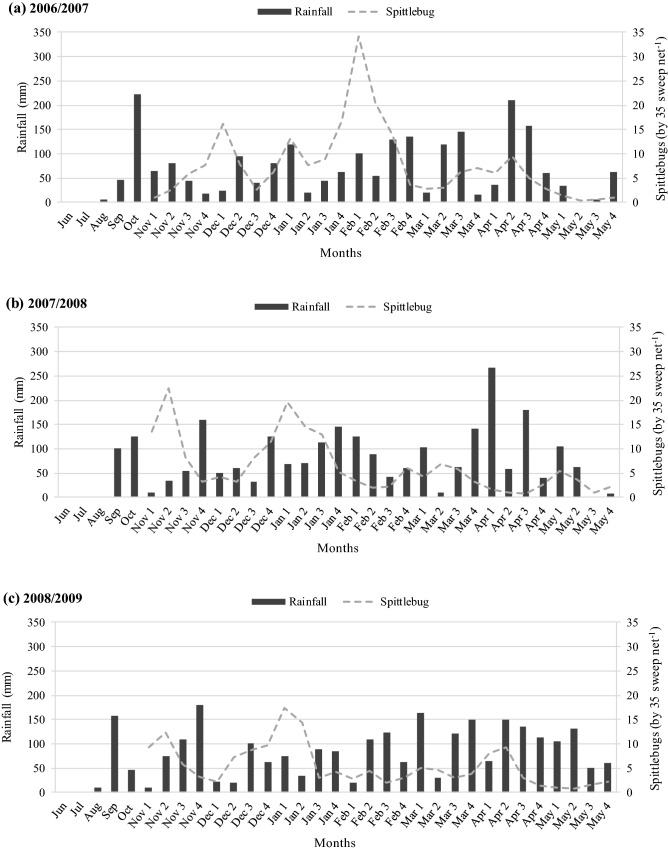
Rainfall and *Mahanarva* abundance and distribution during seasons 2006/2007 (**a**), 2007/2008 (**b**), and 2008/2009 (**c**) at Bernardo Sayão, TO, Brazil. June–October are monthly expressed and November–May are weekly expressed.

Overall, our monitoring suggested the number of generations of *Mahanarva* varied between three and four across seasons (Fig. 2). The spittlebug monitoring resulted in a similar pattern of distribution between seasons; however, the magnitude of peaks within seasons varied. Greater peaks were observed in the 2006/2007 season (34.2 spittlebugs by thirty-five sweep net movements; Fig. 2a) followed by the 2007/2008 (22.5 spittlebugs by thirty-five sweep net movements; Fig. 2b) and the 2008/2009 (17.3 spittlebugs by thirty-five sweep net movements; Fig. 2c) seasons. Consequently, *Mahanarva* abundance followed the same pattern accounting a total of 214.2, 178.5 and 153.6 spittlebugs in 2006/2007, 2007/2008 and 2008/2009, respectively. In contrast, the yearly rainfall accumulated during 2006/2007, 2007/2008 and 2008/2009 seasons were 2,258, 2,505 and 2,664 mm, respectively.

#### Herbage yield

**Figure 3:**
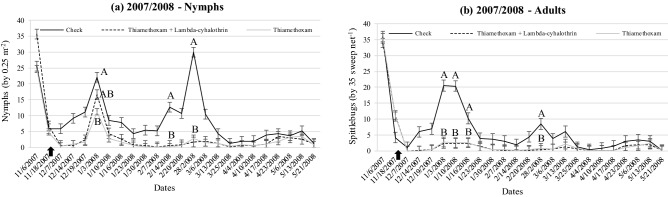
Population density of nymphs (**a**) and adults (**b**) of *Mahanarva* throughout 2007/2008 wet season according treatemnts at Bernardo Sayão, TO, Brazil. Error bars denote the standard error of the mean. Within date, different upper-case letters differ (*P* < 0.05). There was no difference between Thiamethoxam and Thiamethoxam + Lambda-cyhalothrin (*P* > 0.05), then letter B represents both (unless in 1/3/2008 for nymphs). Up arrow indicates the spraying in 11/23/2007.

**Figure 4:**
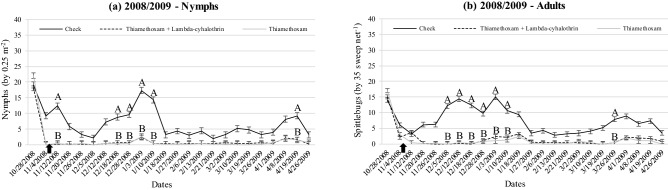
Population density of nymphs (**a**) and adults (**b**) of *Mahanarva* throughout 2008/2009 wet season according treatments at Bernardo Sayão, TO, Brazil. Fertilized and no fertilized were grouped within the same spittlebug control treatment. Error bars denote the standard error of the mean. Within date, different upper-case letters differ (*P* < 0.05). There was no difference between Thiamethoxam and Thiamethoxam + Lambda-cyhalothrin (*P* > 0.05), then letter B represents both. Up arrow indicates the spraying in 11/08/2008.

**Figure 5:**
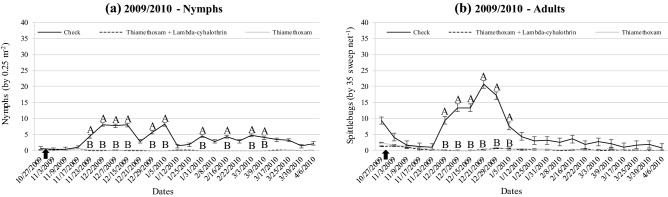
Population density of nymphs (**a**) and adults (**b**) of *Mahanarva* throughout 2009/2010 wet season according treatments at Bernardo Sayão, TO, Brazil. Fertilized and no fertilized were grouped within the same spittlebug control treatment. Error bars denote the standard error of the mean. Within date, different upper-case letters differ (*P* < 0.05). There was no difference between Thiamethoxam and Thiamethoxam + Lambda-cyhalothrin (*P* > 0.05), then letter B represents both. Up arrow indicates the spraying in 11/01/2009.

The spittlebug population density for the field experiments that estimated herbage yield is shown in Figs. 3, 4 and 5. There was an interaction between treatment and date for all wet seasons (*P* < 0.0001). Overall, treatments containing insecticides were effective to keep the populations of nymphs and adults much lower than check (no spittlebug control) during any wet season. The spraying occurred only once at the beginning of each wet season in all experiments.

Herbage yield during 2007/2008 wet season was affected by spittlebug control (*P* = 0.0250; Table 1). The presence of spittlebugs decreased herbage yield about 37% compared to Thiamethoxam (*P* < 0.05) while Thiamethoxam + Lambda-cyhalothrin was similar to both (*P* > 0.05).

**Table 1:** Herbage yield (kg DM ha^−1^) of Marandu palisade grass during 2007/2008 wet season at Bernardo Sayao, TO, Brazil.

Treatments	Herbage yield	SEM	*P* value
Thiamethoxam 75 g a.i. ha^−1^	4,141 A		
Thiamethoxam 56.4 + Lambda-cyhalothrin 42.4 g a.i. ha^−1^	3,643 AB	398.5	0.0250
Check (no spittlebug control)	2,620 B		

Means followed by the same upper-case letter in columns do not differ (*P* > 0.05). Standard error of the mean (SEM).

Herbage yield from 2008/2009 wet season was affected by spittlebug control × fertilization interaction (*P* = 0.0070; Table 2). During the 2008/2009 wet season, non-fertilized spittlebug control treatments presented similar herbage yield than check (*P* > 0.05). However, on fertilized plots, spittlebugs decreased 43% the herbage yield compared with both strategies of control (*P* < 0.05). In contrast, there was no interaction between spittlebug control and fertilization during 2009/2010 wet season (*P* = 0.3114; Table 2). Herbage yield of both Thiamethoxam and Thiamethoxam + Lambda-cyhalothrin averaged 4,669 kg DM ha^−1^, 43% greater than check (*P* = 0.0010). Additionally, there was an effect of fertilization on herbage yield during 2009/2010 wet season (*P* < 0.0001; Table 2).

**Table 2:** Herbage yield (kg DM ha^−1^) of Marandu palisade grass during 2008/2009 and 2009/2010 wet seasons at Bernardo Sayao, TO, Brazil.

Treatments	Fertilization		SEM	*P* value		
	No fertilized	Fertilized		Trt	Fert	Trt*Fert
**2008/2009**						
Thiamethoxam 75 g a.i. ha^−1^	2,126 Ab	12,285 Aa				
Thiamethoxam 56.4 + Lambda-cyhalothrin 42.4 g a.i. ha^−1^	1874 Ab	12,541 Aa	877.8	0.0018	< 0.0001	0.0070
Check (no spittlebug control)	1,570 Ab	7,139 Ba				
**2009/2010**						
Thiamethoxam 75 g a.i. ha^−1^	4,740 A					
Thiamethoxam 56.4 + Lambda-cyhalothrin 42.4 g a.i. ha^−1^	4,597 A		312.6	0.0010	< 0.0001	0.3114
Check (no spittlebug control)	3,256 B					

Within season, means followed by the same upper-case letter in columns and lower-case letter in rows do not differ (*P* > 0.05). In 2008/2009, fertilized corresponded to 1,000 kg ha^−1^ of NPK formula (03-17-00) + 400 kg ha^−1^ of potassium chloride + 500 kg ha^−1^ of urea. In 2009/2010, fertilized corresponded to 300 kg ha^−1^ of NPK formula (03-17-00) + 50 kg ha^−1^ of potassium chloride + 285 kg ha^−1^ of ammonium sulfate. Standard error of the mean (SEM).

#### Beef heifer’s productivity

**Figure 6:**
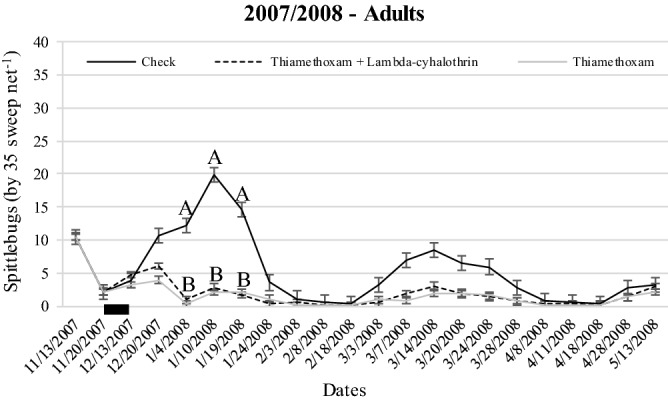
Population density of adults of *Mahanarva* throughout beef heifers experiment (2007/2008) according treatemnts at Bernardo Sayão, TO, Brazil. Error bars denote the standard error of the mean. Within date, different upper-case letters differ (*P* < 0.05). There was no difference between Thiamethoxam and Thiamethoxam + Lambda-cyhalothrin (*P* > 0.05), then letter B represents both. Black bar indicates the window of spraying from 11/20/2007 to 12/13/2007.

The spittlebug population density for the beef heifer’s productivity field experiment is shown in Fig. 6. A single spraying of both Thiamethoxam and Thiamethoxam + Lambda-cyhalothrin was effective to keep the adults’ population of *Mahanarva* significantly lower than check (no spittlebug control) during the most of the 2007/2008 wet season. There was no difference between Thiamethoxam and Thiamethoxam + Lambda-cyhalothrin (*P* > 0.05).

The performance parameters from beef heifers and SR of pastures are presented in Table 3. Average daily gain was not affected by spittlebug control (*P* = 0.2724). However, there was a significant effect of spittlebug control on SR (*P* = 0.0040). Strategies of spittlebug control increased the SR compared to check (*P* < 0.05). In addition, beef heifer’s productivity was greater when Thiamethoxam was adopted as strategy of spittlebug control (*P* < 0.05; Table 3).

**Table 3:** Average daily gain (kg BW day^−1^), stocking rate (animal units ha^−1^), and Nellore beef heifer’s productivity (kg BW ha^−1^) grazing Marandu palisade grass from 2008 January to May at Bernardo Sayao, TO, Brazil.

Treatment	Average daily gain	SEM	*P* value
Thiamethoxam 75 g a.i. ha^−1^	0.38 A		
Thiamethoxam 56.4 + Lambda-cyhalothrin 42.4 g a.i. ha^−1^	0.34 A	0.027	0.2724
Check (no spittlebug control)	0.40 A		

	Stocking rate	SEM	*P* value
Thiamethoxam 75 g a.i. ha^−1^	1.41 A		
Thiamethoxam 56.4 + Lambda-cyhalothrin 42.4 g a.i. ha^−1^	1.01 A	0.185	0.0040
Check (no spittlebug control)	0.48 B		

	Productivity	SEM	*P* value
Thiamethoxam 75 g a.i. ha^−1^	93.2 A		
Thiamethoxam 56.4 + Lambda-cyhalothrin 42.4 g a.i. ha^−1^	51.6 AB	11.36	0.0063
Check (no spittlebug control)	24.4 B		

Means followed by the same upper-case letter in columns do not differ (*P* > 0.05). Standard error of the mean (SEM).

## Discussion

Our spittlebug monitoring across three seasons corroborated with the literature suggesting that number of generations may vary from three to four throughout the wet period^10,12,22^. For the region where the field experiments were carried out, the first rains begun about September/October and the adult spittlebugs increased their abundance from October to November until May when the rains were over. This is the period when cattle producers must be aware in order to monitor the pest abundance and adopt controlling strategies if necessary.

Our results showed that spittlebugs of the *Mahanarva* genus can strongly affect Marandu palisade grass physiology. The greenhouse trial was carried out for twenty days aiming to describe the damage caused by only one generation of adults from *Mahanarva* genus on Marandu palisade grass. It is worth mentioning that the Savannah biome, where most of the Brazilian beef cattle herd is raised, the pastures are grazed throughout an entire wet season (≈ 180 days) exposed to injuries by three to four consecutives generations of nymphs and adults throughout the grazing cycles. At the greenhouse, the herbage regrowth of Marandu palisade grass decreased from 8 to 66% depending on infestation level. The decreased herbage regrowth led a depletion of mineral’s and nutrient’s pools. Pools of Ca, Mg, P, S and K decreased 60%, by average, for the greater infestation level. Nutrients such CP, NDF and IVDDM were decreased by 53, 67 and 66%, respectively. Inferring to field conditions, lower provision of essential minerals and nutrients to the herd would result in reduced animal productivity. These results are in agreement with Valério and Nakano^32–34^ that reported reductions of about 64% on herbage yield and 30% on nutritive value parameters of Signal grass injured by *N. entreriana*. Also in a greenhouse, Taliaferro et al.^27^ and Beck^29^ and reported a decrease on root production and organic reserves caused by *Prosapia bicincta* on Bermuda grass (*Cynodon* sp.). However, these studies were conducted during four-month period mimicking a long-term infestation similar to what would occur during the wet season on field, whilst our greenhouse trial lasted only 20 days.

The results obtained in the greenhouse were corroborated by the field results. *Mahanarva* spittlebugs substantially damaged Marandu palisade grass growth. The losses in terms of herbage yield varied from 37 to 43% during all wet seasons assessed. In the 2008/2009 wet season, where the factor fertilization was included on experiment protocol, the herbage yield responded to a significant interaction between nutrient availability (i.e. fertilization) and spittlebug control. The spittlebugs significantly decreased the herbage yield only where there was fertilization, highlighting that more intensive livestock production systems (i.e. greater inputs) must be more carefully monitored and to adopt strategies of spittlebug control. In the 2009/2010 wet season, when the fertilization level was lower than the one in 2008/2009, the herbage yield of Marandu palisade grass responded both by fertilization and spittlebug control but did not have any interaction. The lack of significance on herbage yield for no fertilized strategies on spittlebug control during 2008/2009 may be explained by a restriction of other abiotic growth factors such as soil moisture or solar radiation, suggested by their reduced herbage production compared to the other wet seasons. On the other hand, Valerio and Nakano^32^ reported greater reductions on herbage yield when Signal grass was exposed to *N. entreriana* under restricted growth factor condition (i.e. soil moisture). The stubble height may have influenced these differences on herbage yield across the wet seasons. Greater severities of grazing or cutting (i.e. lower stubble height) as adopted in 2008/2009 remove more leaf tissue, the major responsible for plant photosynthesis, strongly affecting the subsequent plant regrowth and the yearly herbage production^37^. Lower stubble heights make plant regrowth more dependent on the organic reserve pools and on soil nutrient availability^38^, and the Brazilian Savannah typically presents low soil fertility level.

Several studies have reported reductions on herbage yield of different forage species growing around the world. Wilson et al.^30^ and Parman and Wilson^31^ evaluated alfalfa responses under *Philaenus spumarius* attack and reported decreases of 17 and 20% on herbage yield, respectively. Valério and Nakano^32,33^ reported decreases varying from 34 to 91% caused by *N. entreriana* on Signal grass yield depending on infestation level. Taliaferro et al.^27^ found decreases of 27% on Bermuda grass regrowth injured by *P. bicincta*. In contrast, Resende et al.^39^ reported no decrease on herbage yield of *Brachiaria ruziziensis* infested by sixteen adults of *M. spectabilis* for 6 days; however, the authors found reductions on chlorophyll content and functional plant loss index. The lack of difference on herbage yield in the latter study may be related to short period of infestation. In addition, the magnitude of differences on herbage yield reported in the literature may be due to spittlebug population density, host plant resistance, growth factor availability, and infestation period^20,21,40^.

The strategies of spittlebug control adopted in all of those field experiments were consistently able to keep both nymph’s and adult’s populations of *Mahanarva* lower than check during the main peaks with only one spraying at the beginning of wet seasons. It is mainly due to Thiamethoxam molecule present in different concentrations on both insecticides used. Thiamethoxam is a second-generation neonicotinoid of thianicotinyl subclass that may be uptaken by leaves and roots in the soil, providing a systemic translocation and residual effect^41^. The presence of Thiamethoxam also translocating in the root system ensures an effective control of nymphs^42^. The literature is poor reporting efficacy of spittlebug control strategies for forage species. Recently, Pereira et al.^43^ reported Thiamethoxam as an effective strategy for seed treatment of signal grass to control *D. flavopicta*.

Most literature about pest science for forage species aim to elucidate other questions than the animal productivity losses. In fact, studies about pest science on forage systems are scarce compared to agricultural crops. The little information available has also been focused on plant resistance and genetic traits to support breeding programs^40,44^ and there is a knowledge gap on quantifying animal productivity losses caused by spittlebugs. The quantification of damage caused by pests is one of most important aspects to outline strategies of control and there are no fundamentals to define economic threshold levels with no understanding of the relation between number of insects and crop yield^45^. For agricultural crops, where the final product comes from the cultivated plant, this relation is easier to understand. However, for production systems where what generates revenue is not properly the growing crop but the animals that feed on it, the establishment of economic threshold levels is more complex. In these systems, in addition to the plant and environment components, there is also the animal and the interaction relations that it develops with others, thus determining the final system productivity.

The beef heifer’s experiment was designed to represent an average condition of pasture-based beef production system at the Brazilian Savannah that are usually extensive and composed by Nellore animals grazing Marandu palisade grass. Our results showed a decrease of 74% on beef heifer’s productivity when no strategy of spittlebug control was adopted. The ADG was not affected by spittlebug most likely owing to the criteria used to adjust SR based on daily green leaf allowance of 3 kg DM 100 kg BW^−1^. If the criteria used was based on daily herbage (and not green leaf) allowance, most likely there would be lower herbage quality offered to animals in non-controlled treatment, probably resulting in a decreased performance. The 66% decreased SR in non-controlled treatment was likely led by a reduction of herbage yield of the same magnitude. The results from this field experiment corroborates with the greenhouse trial and other three herbage yield field experiments indicating that Marandu palisade grass is not resistant to spittlebugs from the genus *Mahanarva*.

Our results showed that levels of infestation around 5 nymphs by 0.25 m^−2^ or 10 adults by thirty-five sweep net movements at the beginning of wet season caused losses in terms of beef production of 74%. Based on these findings, we recommend that the ranchers must carefully monitoring the abundance of spittlebugs since the beginning of wet period in order to adopt strategies of control if necessary. It worth mentioning that there is no general strategy of spittlebug control that fits for all farm scenarios. The cattle producers must compare the strategies of control in terms of cost and efficacy, as well as consider aspects such as historic of problems with spittlebugs in the area, spittlebug species and its level of infestation, host pasture specie, and input technology level. As earlier as the cattle producers adopt strategies of control at the beginning of wet season greater will be the financial return of the investment. However, more studies must be outlined in order to address the losses in terms of animal production, only with this information we can define accurately economic threshold levels.

In the scenario where agricultural systems must increase their efficiency in order to meet world's growing demand for food, our findings highlight that spittlebugs may cause significative damage on forage grasses and strongly decrease the animal productivity of pasture-based systems. When protected against *Mahanarva* spittlebugs, Marandu palisade grass increases its provision of essential minerals and nutrients to the animals. Herds with higher provision of nutrients usually result in greater animal performance and shorter growing phase, which reduces their slaughter age, production costs and environmental issues. More efficient livestock systems can increase the provision of animal protein to meet the expected world growing demand for food without deforestation.

## Conclusions

In wet-warm tropical climate, spittlebugs occur during wet season usually throughout three to four generations. Our findings indicate that spittlebugs from the genus *Mahanarva* strongly affect Marandu palisade grass physiology resulting in decreased herbage yield and pools of minerals and nutrients, indicating that this grass cultivar is not resistant to *Mahanarva* spittlebugs. Infestation levels of *Mahanarva* around five nymphs per 0.25 m^2^ or ten adults per thirty-five sweep net movements at the beginning of wet season can reduce in 74% (i.e*.* 93.2 vs 24.4 kg BW ha^−1^) the beef production of Nellore heifers grazing Marandu palisade grass.

## Methods

All procedures for this study were approved by the Animal and Environment Ethics Committees of the University of São Paulo, College of Agriculture “Luiz de Queiroz” (USP/ESALQ). All applicable international, national, and/or institutional guidelines for the care and use of animals were followed.

### Greenhouse trial

The greenhouse experiment was carried out in November 2009 at USP/ESALQ, in Piracicaba, SP, Brazil (22° 42′ S, 47° 38′ W and 546 a.s.l.). One hundred ten pots of 15 dm^−3^ were filled with soil of physical and chemical properties as follows: clay = 198 g kg^−1^, sand = 730 g kg^−1^, silt = 72 g kg^−1^, pH in CaCl_2_ = 4.2, OM = 8 g dm^−3^, P (ion-exchange resin extraction method) = 3 mg dm^−3^, K = 0.5 mmolc dm^−3^, Ca = 12 mmolc dm^−3^, Mg = 6 mmolc dm^−3^, S-SO4 = 5 mg dm^−3^, H + Al = 22 mmolc dm^−3^, sum of bases = 19 mmolc dm^−3^, cation exchange capacity = 41 mmolc dm^−3^, base saturation = 46%. Before seeding, the soil of all pots was individually corrected in order to increase base saturation to 70% and the phosphorus to 30 ppm^46^. Then, all pots were managed under the same cutting and watering regimes by 6 months in order to provide to the plants the condition of an established pasture. Five days previously to treatment’s implementation, all pots received 50 mg dm^−3^ of N and K_2_O (as urea and potassium chloride, respectively) and were homogenized about 15 ± 1-cm height and 42 ± 2 tillers pot^−1^.

Treatments corresponded to five levels of *Mahanarva* infestation (0, 5, 10, 20 and 40 adults pot^−1^) and were allocated to experimental units according to a completely randomized design performing 22 replications per treatment. The species *Mahanarva liturata* (Le Peletier & Serville) and *Mahanarva fimbriolata* (Stål) were collected from elephant grass (*Pennisetum purpureum* Schum.) cultivated pasture, transported to the greenhouse and randomly placed into each pot according to treatment level (i.e. spittlebugs were not sexed prior to infestation). All pots were covered by coarsely woven polyester tulle cages (80 × 50 × 60 cm) to ensure correct infestation level^47^. Every 2 days, insects were collected from the field for replacement of the dead insects to keep the infestation constant during the experimental period^33,34^. The infestation period was 20 days based on the longevity of *M. fimbriolata* adult^48^.

After the infestation period, herbage mass was quantified in two strata. The herbage from soil surface until 15-cm height named as stubble, and above 15-cm height identified as regrowth. Root system was washed and separated from soil using a 1-mm screen. Both herbage and root samples were placed in paper bags, weighed fresh and dried in a forced-air oven at 65 °C to constant weight. Samples were ground through a 1-mm screen (Wiley Mill, Thomas Scientific, Philadelphia, PA, USA) to determine chemical composition. Dry matter (DM) and ash were determined at 105 °C for 24 h and 600 °C for 4 h, respectively^49^. Samples from regrowth were analyzed for phosphorus (P), potassium (K), calcium (Ca), magnesium (Mg) and sulfur (S) mineral contents according to Sarruge and Haag^50^. Neutral detergent fiber (NDF) was determined by fiber analyzer (ANKOM Technology Corp., Macedon, NY, USA), described by Holden^51^. Total nitrogen (N) content was determined by the Dumas combustion method using N analyzer (Leco Instruments Inc., St. Joseph, MI, USA), and crude protein (CP) calculated as N × 6.25. In vitro digestible dry matter (IVDDM) was determined by the two-stage procedure of Tilley and Terry^52^ modified by Goering and Van Soest^53^. Both stubble and root samples were only analyzed in terms of organic reserves (total N and non-fibrous carbohydrate (NFC)), and NFC was determined according Passos et al.^54^. To obtain the pool of nutrients, each component mass was multiplied by its respective nutrient content.

### Field experiments

**Figure 7:**
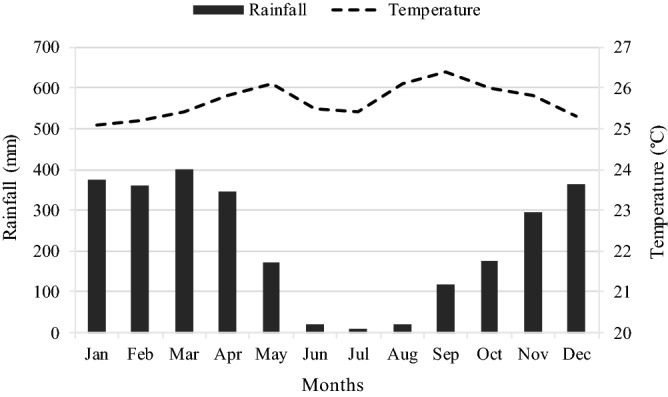
Average rainfall and temperature at Bernardo Sayão, TO, Brazil (1999–2009).

The field experiments were carried out at Terra Grande Ranch (8° 5′ S, 48° 58′ W and 177 a.s.l.) in Bernardo Sayão, TO, Brazil, on a rainfed Marandu palisade grass {*Brachiaria brizantha* (Hochst.ex A. Rich.) R. D. Webster [syn. *Urochloa brizantha* (A. Rich.) Stapf]} pasture established in a Plinthosol^55^ with average chemical properties as followed: pH in CaCl_2_ = 4.5, OM = 16.3 g dm^−3^, P (ion-exchange resin extraction method) = 3 mg dm^−3^, K = 0.7 mmolc dm^−3^, Ca = 9.2 mmolc dm^−3^, Mg = 3.3 mmolc dm^−3^, S-SO4 = 11.5 mg dm^−3^, H + Al = 34.9 mmolc dm^−3^, sum of bases = 13.4 mmolc dm^−3^, cation exchange capacity = 48.2 mmolc dm^−3^, base saturation = 25.9%. The Terra Grande Ranch was chosen as the place to carry out the field experiments because of its historical problem with spittlebug infestations and because it had an average condition of animal productivity from beef production systems in the tropics (i.e. average productivity of 80–120 kg BW ha year^−1^). The climate is Aw moist-warm tropical according Köppen, with accumulated annual rainfall and mean temperature of 2,660 mm and 25.7 °C, respectively. The first rains begun in September and finished in May, with the greatest rainfall recorded from November to April (Fig. 7).

The spittlebug distribution and abundance were weekly monitored during three consecutive rainy seasons (2006/2007, 2007/2008, 2008/2009) aiming to describe the variation pattern and the number of generations within and between seasons. The monitoring was carried out in Marandu palisade grass paddocks with predominance of spittlebugs from the genus *Mahanarva*. The insects were monitored by eight sweep net (50 cm diameter) sub-sampling composed by thirty-five movements each across the paddock. The predominance of *Mahanarva* spittlebugs over other genus was found greater than 90%, based on visual assessments of the sweep net after samplings.

There were conducted three field experiments during consecutive rainy seasons (2007/2008, 2008/2009 and 2009/2010) aiming to estimate the herbage yield of Marandu palisade grass damaged by spittlebugs from the genus *Mahanarva*. The specific information about each experiment was described in Table 4. Before treatment implementation, the experimental areas were mowed to 10-cm height for standardization. The treatments containing insecticides were sprayed by tractor equipped with a rod sprayer (Condor Pec 600 M12, Jacto, Pompeia, SP, Brazil) containing eight flat fan nozzles (XR 110.02; Teejet Technologies, Springfield, IL, USA) adjusted to spray 200 L ha^−1^. The fertilizer was applied with a Vincon PS 603 spreader (Vincon Maquinas Agricolas LTDA, Cotia, SP, Brazil). The spittlebug infestation was monitored weekly and the sward height twice per week. The sward height was measured from ground level to the top leafy horizon using a stick graduated in centimeters^56^. For all experiments and treatments, the herbage mass was harvested when Marandu palisade grass reached 35-cm height^57^, and the stubble height varied among experiments (Table 4). Herbage samples were dried as in the greenhouse trial. For all of the field experiments, treatments were allocated to experimental units (plots) according to a randomized complete block design, with five replications.

**Table 4:** Specific information of each of three experiments conducted during 2006/2007, 2007/2008 and 2008/2009 wet seasons at Bernardo Sayao, TO, Brazil.

Season	Experimental unit (m^2^)	Useful area (m^2^)	Stubble height (cm)	Treatments	Sampling procedures
2007/2008 Nov–May 190 days	700	30	15	1- Thiamethoxam 75 g a.i. ha^−1^ 2- Thiamethoxam 56.4 + Lambda-cyhalothrin 42.4 g a.i. ha^−1^ 3- Check (no spittlebug control)	Sward height: 25 readings Herbage mass: two sub-samples of 1 m^2^ each Adult: two sweep net sub-samples composed by thirty-five movements each Nymphs: two sub-samples of 0.25 m^2^ each
2008/2009 Jan–May 140 days	3,600	400	5	1- Thiamethoxam 75 g a.i. ha^−1^ 2- Thiamethoxam 75 g a.i. ha^−1^ + fertilization 3- Thiamethoxam 56.4 + Lambda-cyhalothrin 42.4 g a.i. ha^−1^ 4- Thiamethoxam 56.4 + Lambda-cyhalothrin 42.4 g a.i. ha^−1^ + fertilization 5- No spittlebug control + fertilization 6- Check (no spittlebug control + no fertilization)	Sward height: 50 readings Herbage mass: four sub-samples of 1 m^2^ each Adult: four sweep net sub-samples composed by thirty-five movements each Nymphs: four sub-samples of 0.25 m^2^ each
2009/2010 Nov–Apr 155 days	3,600	400	20	1- Thiamethoxam 75 g a.i. ha^−1^ 2- Thiamethoxam 75 g a.i. ha^−1^ + fertilization 3- Thiamethoxam 56.4 + Lambda-cyhalothrin 42.4 g a.i. ha^−1^ 4- Thiamethoxam 56.4 + Lambda-cyhalothrin 42.4 g a.i. ha^−1^ + fertilization 5- No spittlebug control + fertilization 6- Check (no spittlebug control + no fertilization)	Sward height: 50 readings Herbage mass: four sub-samples of 1 m^2^ each Adult: four sweep net sub-samples composed by thirty-five movements each Nymphs: four sub-samples of 0.25 m^2^ each

Useful area represents the area located at the center of the experimental unit where samples were taken. In 2008/2009, fertilization corresponded to 1,000 kg ha^−1^ of NPK formula (03-17-00) + 400 kg ha^−1^ of potassium chloride + 500 kg ha^−1^ of urea. NPK was spread once before experiment begin while potassium chloride and urea were equal split in five installments throughout experiment. In 2009/2010, fertilization corresponded to 300 kg ha^−1^ of NPK formula (03-17-00) + 50 kg ha^−1^ of potassium chloride + 285 kg ha^−1^ of ammonium sulfate. NPK was spread once before experiment begin while potassium chloride and ammonium sulfate were split at the beginning of experiment.

Another field experiment was carried out from November 2007 to May 2008 on Marandu palisade grass sward infested by spittlebug from the genus *Mahanarva*. The objective was to estimate the losses in terms of beef productivity from a typical pasture-based system in the Brazilian tropic. The three treatments were (1) Thiamethoxam 75 g of active ingredient (a.i.) ha^−1^, (2) Thiamethoxam 56.4 + Lambda-cyhalothrin 42.4 g a.i. ha^−1^ and (3) Check (no spittlebug control). Treatments were allocated to experimental units (13 ha paddocks) according to a randomized complete block design, with four replications. The slope, chemical soil characteristics and sward condition were considered as blocking criteria. Before treatment implementation, the paddocks were standardized at 15-cm height using heifers from the ranch herd. The spraying was performed with the same equipment and volume aforementioned. The spittlebug infestation level was monitored by eight sweep net (50-cm diameter) sub-sampling composed by thirty-five movements each alongside each paddock. Pastures were continuously stocked using a variable SR (i.e. put-and-take)^58^. One hundred-eight Nellore beef heifers averaging 270 kg BW and 26 months were stratified according BW, and then randomly assigned to the treatments. These animals were considered as testers and were used to estimate the ADG every 28 days after fasting of 14 h^4^. Put-and-take animals with similar characteristics as the tester heifers were used to adjust the SR. The SR was adjusted every 14 days in order to provide a daily green leaf allowance of 3 kg DM 100 kg BW^−1^ considering the testers and put-and-take animals. The herbage mass was estimated through 5 sub-samples per paddock harvested from soil surface level^4^ and plant-part components by hand separation into leaf (leaf blades), stem (stems + leaf sheaths) and dead material^56^. The average SR, expressed as animal unit (450 kg BW) ha d^−1^, was calculated by multiplying days of grazing for each tester and put-and-take animal on a pasture by the average weight of that animal during the experiment, summing this product across all animals in the paddock, and then dividing by 450 and by the number of grazing days for that pasture^59^. The animal productivity, expressed as kg of BW ha^−1^, was estimated as the product of tester ADG and average SR.

### Statistical analysis

Regression and variance analyses were performed using the GLIMMIX Procedure of SAS (SAS 9.3; SAS Institute Inc., Cary, NC). Different structures of the variance–covariance matrices were tested, and the Bayesian Information Criterion was adopted to select the best fit matrix^60^. For greenhouse trial, treatments were allocated according to a completely randomized design (n = 22), pots were considered experimental units and treated as random terms, and infestation level considered as fixed effect. Herbage yield and beef productivity experiments were set according to a randomized complete block design (n = 5 and n = 4, respectively), plots and paddocks were treated as experimental units and were considered random terms^61^. Variables presented Gaussian distribution and identity link function. Strategies of spittlebug control, fertilization (2008/2009 and 2009/2010 herbage yield field experiments) and their interactions were treated as fixed effects. To analyze the population density of both nymphs and adults during field experiments, date was considered fixed effect. Means were calculated using the Least-Squares Means statement and compared using the Tukey–Kramer test at significance level of *P* ≤ 0.05.

## Data Availability

The datasets generated during and/or analyzed during the current study can be available from the corresponding author upon request.
